# Vaccine Prophylaxis of Pneumococcal Infections in Children under Conditions of Severe Flood in the Amur River Basin

**DOI:** 10.1155/2019/5467275

**Published:** 2019-02-18

**Authors:** Aleksandr G. Chuchalin, Gennadiy G. Onishchenko, Victor P. Kolosov, Olga P. Kurganova, Tatiana A. Zaitseva, Leonid G. Manakov, Galina N. Kholodok, Juliy M. Perelman, Roman S. Kozlov, Natalia M. Ivakhnishina, Olga E. Trotsenko, Albina P. Bondarenko

**Affiliations:** ^1^Department of Hospital Therapy, Pediatric Faculty, Pirogov Russian National Research Medical University, Moscow 117997, Russia; ^2^Russian Academy of Sciences, Moscow 119991, Russia; ^3^Laboratory of Prophylaxis of Nonspecific Pulmonary Diseases, Far Eastern Scientific Center of Physiology and Pathology of Respiration, Blagoveshchensk 675000, Russia; ^4^The Board of the Federal Service for Surveillance in Protection of Consumer Rights and Human Well-Being in the Amur Region, Blagoveshchensk 675002, Russia; ^5^The Board of the Federal Service for Surveillance in Protection of Consumer Rights and Human Well-Being in Khabarovsk Territory, Khabarovsk 680009, Russia; ^6^Research Institute of Protection of Maternity and Childhood, Far Eastern Scientific Center of Physiology and Pathology of Respiration, Khabarovsk 680022, Russia; ^7^The Smolensk State Medical University, Smolensk 214019, Russia; ^8^The Khabarovsk Research Institute of Epidemiology and Microbiology, Khabarovsk 680610, Russia

## Abstract

**Background:**

Pneumococcal infection being one of the dominant causes of acute respiratory diseases and exacerbation of chronic ones is a serious problem for human health and society. The flood in the Amur river basin in the summer of 2013 created a special zone and risk conditions for the formation of respiratory pathology in the Far-Eastern region of Russia. We aimed to give clinical and epidemiological assessment of the effectiveness of vaccination programs of respiratory viral and pneumococcal infections and generalization of regional experience in the organization of a set of measures aimed at their prevention in the postflood period in the Far-Eastern region.

**Methods:**

The monitoring program includes children aged 2 to 5 years in the amount of 4988 with risk factors for pneumococcal infection. The pneumococcal conjugate vaccine* Prevenar-13* was used for immunization. Data on the incidence of ARVI and pneumonia in children in pre- and postvaccination periods were to be recorded. The indicators and special criteria were used to assess the effectiveness of vaccination. To study the circulation of serovariants of pneumococcus in inflammatory diseases of the respiratory tract and nasopharyngeal carrier, bacteriological and molecular genetic methods (RT-PCR in the mode of multiprime detection) were used.

**Results:**

Differences in the frequency and range of serovariants of circulating isolates of pneumococcus in the postvaccinal period and in unvaccinated children, elimination of a number of serotypes, and appearance of circulation of nonvaccinated strains were revealed. The incidence of acute respiratory diseases and pneumonia among the vaccinated population for 2 years in the region decreased by 2.5 times. The coefficient of effectiveness of vaccination according to the indicator of morbidity of children with pneumonia reaches 75-100% with direct dependence on the age of children (r=0.98).

**Conclusion:**

Comparative statistical analysis revealed a high degree of effectiveness of regional programs with the methods of immunoprophylaxis of pneumococcal infections.

## 1. Introduction

Pneumococcal infection being one of the dominant causes of acute respiratory diseases and exacerbation of chronic ones is a serious problem for human health and society. In the Russian Federation, pneumonia ranks first among the causes of mortality from infectious diseases and sixth among all causes of mortality and causes a high level of socioeconomic losses for society and the state. Pneumococcal infections, being widespread diseases, affect people of any age, while children, elderly people, and those with chronic diseases are most at risk of disease [1–4].

Currently, pneumonia as an independent infectious nosological form is of interest from the point of view of epidemiologists as a group of infections that are spreading fast among the population and are characterized by the peculiarities of the manifestations of the epidemic process, by the specificity of pathogens and as the ones that have a certain epidemic potential. Consequently, pneumonia as an infectious pathology needs targeted epidemiological surveillance and the development of an adequate set of sanitary and antiepidemic and preventive measures [5–8].

Along with the known risk factors for pneumonia, climatic conditions are important for the formation of pathological conditions of the respiratory system [9, 10]. The results of epidemiological analysis indicate higher (in comparison with other regions) levels of prevalence of pneumonia in the Far-Eastern Federal district, the peculiarity of which is the extreme climate manifestations of climatic factors that have adverse effects on the respiratory system [11, 12]. The flood in the Amur river basin in the summer of 2013 created a special zone and risk conditions for the formation of respiratory pathology in the Far-Eastern region. On the territory of the Amur region, 22 of 28 municipalities, 126 settlements, and 7,444 residential houses with a population of more than 127,460 people, including 10,015 children, were flooded. These circumstances led to a high risk of infectious diseases, including pneumonia, acute respiratory infections, especially among children and the elderly, and the need to organize urgent measures for their prevention [13].

Specific vaccine prophylaxis is the most affordable and economically justified way to influence the incidence of pneumococcal infections, primarily in the risk groups [5, 14, 15]. In order to prevent influenza, ARVI and community-acquired pneumonia, pneumococcal, and influenza vaccines are used in Russia. In 2014, vaccination against pneumococcal infections is included in the national calendar of preventive vaccinations of the Russian Federation for children of the 1st year of life and in the calendar of vaccinations for epidemic indications [16, 17]. Currently, in accordance with the recommendations for the prevention of pneumococcal infection, both polysaccharide and conjugate vaccines are used [18, 19]. The European Medical Association (EMA), WHO, the Center for Disease Control and Prevention in the United States (CDC), and the Russian Respiratory Society recommend starting vaccination against pneumococcal infection with pneumococcal 13-valent vaccine Prevenar-13 (PCV-13) which provides high immunogenicity and long-term effective protection against diseases caused by* S. pneumoniae* serotypes significant on the territory of the Russian Federation [20–22].

The expert community noted that in Russia and abroad there is enough data on the clinical effectiveness of vaccination of children and adults at risk and its high economic efficiency was demonstrated [2, 15, 23]. At the same time, the use of antipneumococcal vaccines is becoming a standard international and Russian practice recommended for use in regional health programs.

In this regard, the* aim* of the study was to conduct a clinical and epidemiological assessment of the effectiveness of vaccination against respiratory viral and pneumococcal infections and summarize regional experience in the organization of a set of measures aimed at their prevention in the postflood period in the Far-Eastern region of Russia.

## 2. Materials and Methods

The surveyed people included in the program of monitoring and clinical and epidemiological assessment of the effectiveness of vaccination are represented by children aged 2 to 5 years in the number of 4988 with risk factors for pneumococcal infection. Vaccination of children was done in March-May 2014 in 17 municipalities of the Amur region (coverage was 22.1% in this age group and 85-90% in the risk group). Vaccinated children were represented almost in equal proportions by age groups from 2 to 3 years, from 3 to 4 years and from 4 to 5 years.

For immunization against pneumococcal infections, pneumococcal conjugate vaccine Prevenar-13 was used. In 13.1% of cases, vaccination was done in combination with the use of an anti-influenza vaccine. Modes of administration, dosage, and conditions of use of the vaccine were done in accordance with Federal Clinical Guidelines [16, 17] and Instructions for use of the vaccine.

During the implementation of the program activities, the effectiveness of vaccination for specific prevention of pneumococcal infections was assessed using the methods of clinical and epidemiological analysis and economic and statistical analysis. This was done using the database of the regional health administration bodies, bodies of state statistics of Russia (Rosstat).

The main indicators of the effectiveness of vaccination against pneumococcal infection (in addition to the incidence of infectious diseases) were chosen to be the following: the total duration of the disease, the number of courses of antibacterial therapy and the frequency of hospitalization in pre- and postvaccination periods. To assess the effectiveness and quality of specific prevention, special criteria were used: the coefficient of preventive effectiveness (CE) of vaccination and the infectious index (II).

As part of the epidemiological monitoring of pneumonia, the analysis of the main characteristics of the epidemic process, of the dynamics of morbidity, of its seasonality, of the age structure of patients, and of the etiology of the disease was done. In the analysis of the social and epidemiological effectiveness of vaccination, the impact of pneumococcal vaccination on the incidence of pneumonia among the total population (children aged 0 to 14 years), both those caught in the flood zone and those free from it over a number of years, was assessed.

In accordance with the program, monitoring studies were done to assess the circulation of serological variants of* S. pneumoniae* in postflood period on the territory of Khabarovsk Krai. To assess the molecular genetic characteristics of* S. pneumonia* and to identify the effectiveness of vaccination against pneumococcal infection in the postflood period on the Amur river, healthy children under the age of 5 years (Khabarovsk, 2014) were examined; they visited preschool institutions that were in the flood zone before and a year after vaccination. All studies were approved by the Local Ethics Committee and were conducted after informed parental consent.

At the stage of studying the molecular genetic structure of pneumococci to assess the circulation of serovariants of pneumococcus in inflammatory diseases of the respiratory tract and nasopharyngeal carrier by RT-PCR in the mode of multiprime detection, strains of* S. pneumoniae* were tested (n=57), isolated in children carriers (n=22), in patients with pneumonia (n=15), and in those with noninvasive nasopharyngeal infection (n=20). In order to identify the phenotypic features and genetic characteristics of* S. pneumoniae*, the strains isolated from the carriers, patients with community-acquired pneumonia, and acute sinusitis (n=363) were also studied.

The material was studied by the bacteriological methods (64 samples) and molecular genetic (RT-PCR in the mode of multiprime detection) methods (94 samples). The samples from the nasopharynx were taken by using sterile swabs which were later inoculated in 0.5 ml of the “Transport medium for storage and transport of respiratory smears” (OOO “InterLabService” of FBIS CNIIE of The Russian Federal Service for Surveillance on Consumer Rights Protection and Human Well-being”) in an Eppendorf test-tube. The identification of microorganisms was performed by standard methods with the use of the certified culture media (Mueller agar, Hinton agar, BioMerieux agar) and reagents (BioMerieux, BioRad, Lachema).

The obtained samples from pure cultures (57) and samples from the primary material (nasopharynx washing) (54) were tested to reveal DNA of* S. pneumoniae* by the RT-PCR method with genes lytA and psaA as targets. The extraction of DNA of* S. pneumoniae* from the bacterial suspension was done according to the instruction attached to the reagents *«*DNA-sorb-AM” made by the company “InterLabService”. The RT-PCR was performed on the thermal cycler CFX 96 (“BioRad”, USA) in the multiprime mode, with the volume of 25 *μ*L. For serotyping of* S. pneumoniae* the primers recommended for serotyping by the Center for Disease Control and Prevention (CDC, USA) prepared at the Central Research Institute of Epidemiology of the Russian Federal Service for Surveillance on Consumer Rights Protection and Human Well-being were used. The multilocus sequencing-typing was performed in 13 clinical strains of pneumococci per protocols of MLST and CDC. The sensitivity to antimicrobial drugs (AMD) was determined by the method of serial dilutions, used according to the standard CLSI M100-S24E, 2014 within the framework of the project “PeGas IV”.

The analytical stage of evaluation of efficiency of the program included statistical processing of primary materials on the basis of the Microsoft Excel 2010 program package with calculation of the main indicators (relative sizes, errors of reliability of differences of indicators, and conjugation of signs). Methods of mathematical statistics (pair correlation, regression, and comparative analysis) were used for the analysis and evaluation of statistical material.

## 3. Results and Discussion

### 3.1. Etiology of Community-Acquired Pneumonia and Postvaccination Dynamics of Molecular Genetic Variants of Isolates of* S. pneumoniae* in Children of the Amur River Region

**Figure 1:**
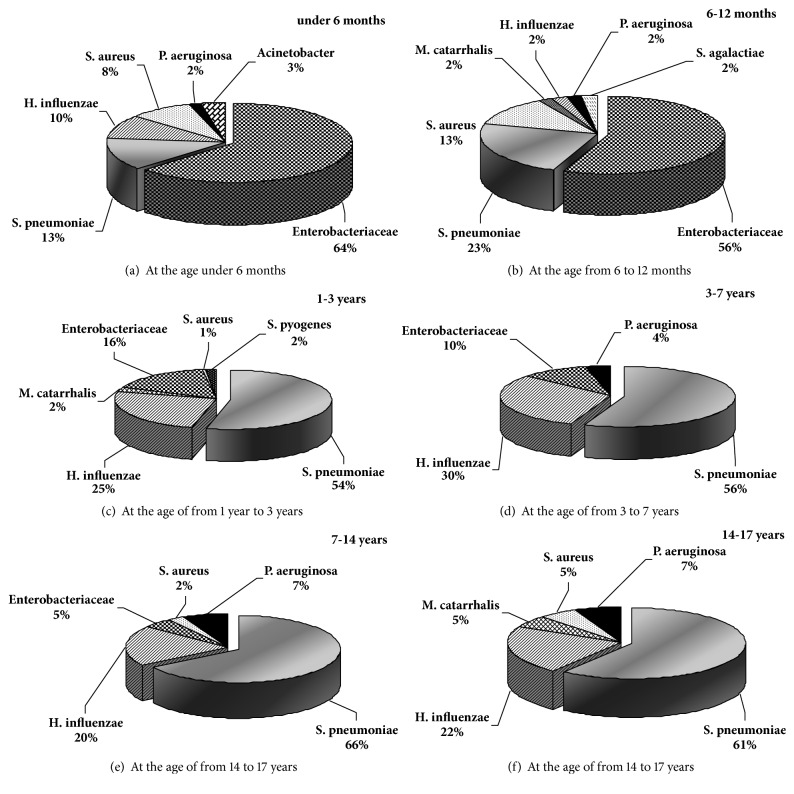
Etiology of pneumonia in different age groups of children (Khabarovsk Territory, %).

Bacteriological studies (n=811) allowed establishing the etiology of community-acquired pneumonia in children, in which the leading role belongs to pneumoniae. The frequency of its isolation from the tracheal aspirate is 13.3-23% in children of the first year of life; 51.8-65.9% in children over 1 year (Figure 1). In general, positive seeding of pneumotropic microorganisms in diagnostic titers (5-6 lg CFU/ml of aspirate and more) was obtained in 306 patients (37.7%).

In monoculture* S. pneumoniae* was isolated in 90% of cases. A coinfection was found in 10%. In 67% cases it was caused by associations of* S. pneumoniae* with* H. influenzae* (32%),* Enterobacteriaceae* (25%), and* M. catarrhalis* (10%). In 33% cases there was a combination of* H. influenzae* (NTHi and Hib) with* Enterobacteriaceae* and with* Acinetobacter spp.* The detection rate of* H. influenzae* was 6.9%, predominantly in the composition of coinfections with* S. pneumoniae*. The detection of Hib strains accounted for 0.4% cases (5.6% of all clinical strains of* H. influenzae*). The significant differences in the microbial spectrum of the tracheal aspirate in children were established depending on the age: prevalence of the detection rate of* Enterobacteriaceae *(64-56%) in the first and rarely in the second half of the first year of life and of* S. pneumoniae* and* H. influenzae* after the 1st year of life.

Analysis of clinical strains (isolates 2012-13) of 7 genes of the main metabolism, the aro gene (shikimate dehydrogenase) in comparison with the same genes of Chinese, American, and English strains by multilocus sequencing (MLST), showed their greatest similarity with the Chinese strains A026, ST556 (a number of genes revealed 100% identity) [24, 25]. The similarity with strains revealed by American and English researchers TCH8431/19A, Taiwan19F-14, 458.1JA00S, 544.1Tx99S, 687.1CH99S, SPN994038, 0.45SL99S, and ATCC 700669 was also found. Consequently, phylogenetic pneumococcus has a high probability of origin from a common ancestor with strains from China, America, and the UK.

According to molecular genetic studies, 8 serovariants/groups of* S. pneumoniae* were identified, 87.5% of which belong to the vaccine strains included in the conjugate vaccine Prevenar-13 and 73.7% are basic variants which are of clinical significance in Russia: 19F, 6AB, and 3. Serovariant 11 AD isolated from the carrier did not belong to the vaccine strains. In 14% (8 out of 57) the serotype was not established (untyped strains).

The revealed serovariants were present in all studied groups: both in carriers and in those with the respiratory pathology. Clinically significant vaccine serovariant 19F was found 2 times more often in the respiratory pathology: in 53% of cases in community-acquired pneumonia, in 50% in sinusitis, and 22.7% in carriers (p=0.0606). Serogroup 6AV was detected in carriers with noninvasive pneumococcal infection in 22.7% of cases, which was 1.9 times more often than in pneumonia. On the contrary, serotype 3, which causes severe pneumonias, including destructive ones, was detected with high frequency in pneumonia (20%) and quite often (13%) in carriers. Serotypes 19F and 3 were detected mainly in patients with community-acquired pneumonia; 6AV was in carriers and in those with a noninvasive infection (sinusitis).

Thus, the established range of serological variants of* S. pneumoniae* in carriers and patients with respiratory pathology in the city of Khabarovsk was characterized by a predominance of 3 options: 19F, 6AB, and 3, making 73.7% of all the obtained strains. All isolates belonged to the vaccine strains included in the pneumococcal conjugate vaccine Prevenar-13. The coincidence of the established range of pneumococcus with vaccine strains showed the effectiveness of scheduled vaccination and reduction of nosocomial carriage of* S. pneumoniae* and of the incidence of pneumococcal infections in children.

An increase in resistance to antimicrobial drugs (AMD) has been established. Of the 68 clinical strains of* S. pneumoniae*, 86.8% are sensitive to amoxicillin, 7.4% are resistant, and 5.8% are moderately resistant. 85.3% are sensitive to penicillin, only 1.5% of strains are resistant, and 13.2% are in the range of moderate resistance. Ceftriaxone is active in 73.5% of cases; resistance was revealed in 22%. The greatest resistance was established to macrolides, mainly to azithromycin (61%) (MPC_50-90_-64 mg/l), to tetracycline (65%), and to trimethoprim (69%), respectively. Polyresistance was revealed in 51% of cases. The established levels of resistance of strains of* S. pneumoniae* isolated from sputum/bronchoalveolar lavage in children with community-acquired pneumonia exceed those recorded in the western regions of Russia.

Thus, almost all strains circulating in the studied child population are vaccine serovariants, including 19F, 14, 6 A/B/C/D, 4, 23F serogroups and serotype 3, with a predominance of serotypes 19 F, which are associated with resistance to AMD. According to the screening results of the resistance of pneumococci isolated from children in the kindergarten, using a disc with oxacillin 1 *μ*g/disc (taking into account EUCAST standards), 64% of serotype 19F strains were resistant to *β*-lactam antibiotics.

**Figure 2:**
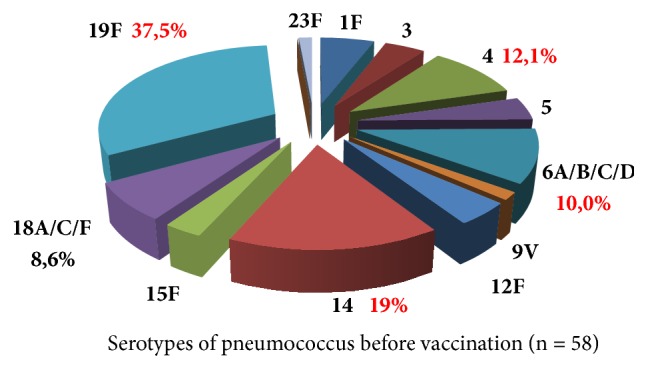
Spectrum of serovariants of* S. pneumoniae* in carriers before vaccination with* PCV-13* (Khabarovsk Territory, 2014, %).

The evaluation of nasopharyngeal carriage of pneumococcus among children in kindergarten showed that before vaccination the range of serovariants of pneumococcus included all vaccine strains, except for 7F and 19A, with a predominance of the circulation of serotype 19F (37.5%). Serotype 14 was detected in 19%, serotype 4 in 12.1%, and 6A / B/C / D in 10% (Figure 2).

Emergency vaccination against pneumococcal infection in the region has led to a change in the range of serovariants with the elimination of a number of invasive and resistant populations of pneumococcus. At the same time the predominance of nasopharyngeal carriage of 19F serovariant remained, but a circulation of nonvaccine strains that do not have clinical significance appeared.

**Figure 3:**
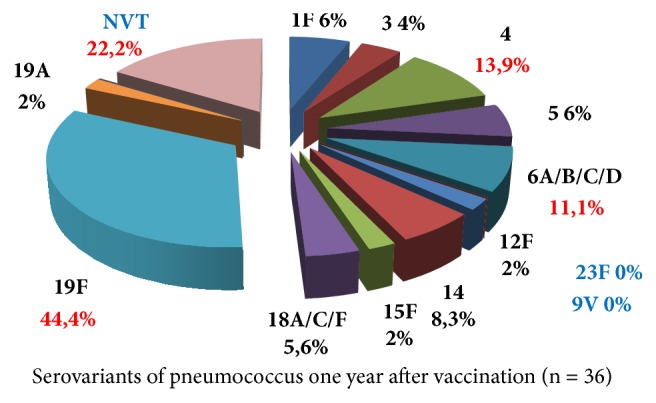
Serological variants of* S. pneumoniae *1 year after vaccination with PCV-13 in the examined group (Khabarovsk Territory, %).

A year after vaccination (n=36), the main range of serotypes remained the same with a predominance of 19F serotype (44.4%). Serotypes 23 and 9V and nonvaccine serotypes 11A/D, 12F/A, and 15A/F were eliminated. A reduction in the carriage of serogroups 14 from 19 to 8.3% and serogroups 18A/C/F from 7 to 5.6% was found out. Some vaccine strains were replaced by nonvaccine strains. Nonvaccine strains (NVT) were found in 22.2% of cases. Full replacement of serovariants was found in 38.9% of children. The dynamics of the change of serovariants is shown in Figure 3.

Among serovariants eliminated after vaccination at the individual level, almost all circulating in the study population options were revealed. It was reliably determined that only vaccinated children a year later in 32.1% of cases had no nasopharyngeal carrier of pneumococcus (p<0.05), which proves the effectiveness of vaccination. Carriage of prevaccination serovariant persisted in 61.1% of cases, out of these at persistence of the previous variant after vaccination in 22.2% of children (8 out of 36) the appearance of carriage of additional vaccine product occurred. According to the results of the survey, it was found that in a year in the nonvaccinated children (n=23) the frequency of carriage of serovariant 19F increased almost twice and amounted to 62.2% (p<0.05), displacing other serovariants from the population. Thus, in the absence of vaccination, the increase in the population of serotype 19F of pneumococcus is dangerous from the viewpoint of the spread and increase of resistance of pneumococcus to antimicrobial drugs.

### 3.2. Epidemiology of Pneumonia, Organization, and Clinical and Epidemiological Assessment of the Effectiveness of Preventive Programs in the Region

**Figure 4:**
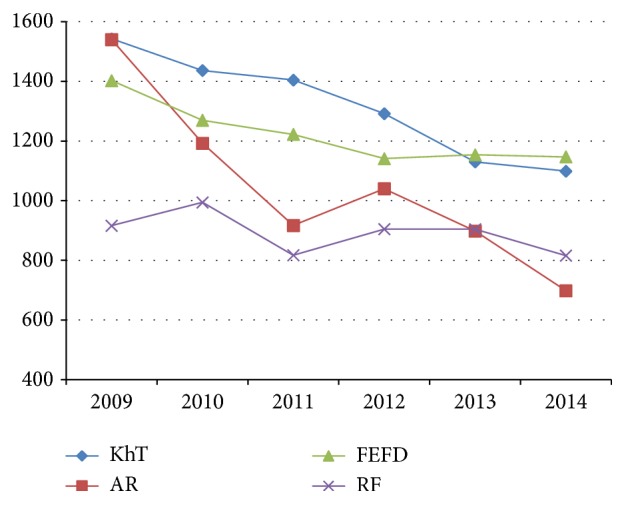
Dynamics of pneumonia morbidity of children's population on the territory of the Far East Federal District (per 100 000 children's population); KhT: Khabarovsk Territory, AR: Amur Region, FEFD: Far East Federal District, and RF: Russian Federation.

The analysis of dynamics of indicators of incidence of pneumonia of children on the territory of the Amur region and Khabarovsk Krai testifies (Figure 4) that its peak incidence reached in 2009, which was associated with the influenza pandemic. At the same time, there is a strong correlation between the intra-annual dynamics of pneumonia incidence and the level of ARVI incidence (r=0.89, t≥2.0, at p≤0.05), which allows for preventive measures with a wide range of effects on the epidemic process. In the subsequent period (2010-2014) there was a decrease in morbidity by 54.7% (Amur region) and 28.8% (Khabarovsk Krai).

The structure of patients with pneumonia is dominated by an adult population making 75.0±0.45% (average for 2010-2015). However, the intensive parameters of the incidence are much higher among children and adolescents (854.1±114.2 per 100 000 of the respective age groups) than among the adult population (609.1±59.8 per 100 000), at p≤0.05. It was found out that the disease is most susceptible to children of younger age group (0 to 2 years) and the elderly (over 65 years). Incidence rates among those age groups are, respectively, 1732.0 and 1303.0 per 100 000 population of corresponding age with minimal morbidity among persons in the age group of 18-39 years (460.0:100 000).

**Figure 5:**
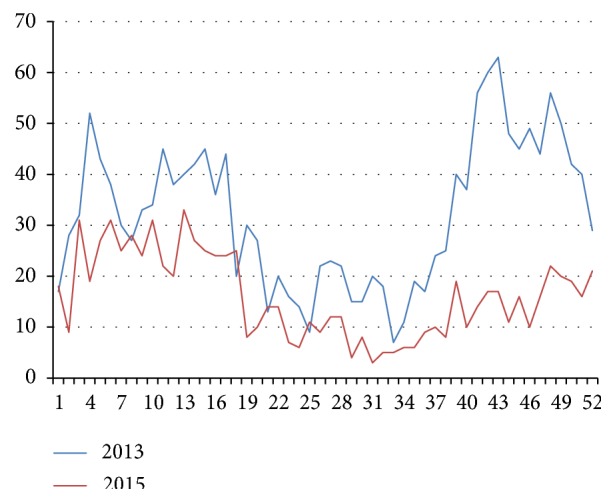
Intra-annual (week by week) dynamics of pneumonia morbidity of children's population of the Amur Region (epidemiological monitoring, absolute values).

In the analysis of the annual dynamics of the incidence of pneumonia there is a pronounced seasonality (Figure 5). It was found out that during the year two periods of morbidity rise are registered: from February to April (with the maximum number of registered cases at weeks 4-7) and from September to December (with the maximum number of registered cases at weeks 42-50). The peak of morbidity rates is observed in March and October with an average annual value of the indicator for 2010-2015–654.6±53.2 per 100 000 of the total population.

Analysis of indicators of the effectiveness of vaccination against pneumococcal infection shows that in the postvaccination period the total duration of the disease decreased by 14.6%; the number of courses of antibacterial therapy (ABT) decreased by 21.3% compared to the prevaccination period. However, the most significant positive changes in the indicator were observed with respect to the frequency of hospitalization of patients, which in the postvaccination period in most municipalities decreased by 2-3 times (p=0.0389).

Comparative statistical analysis of the effectiveness of vaccination against pneumococcal infections with the use of the vaccine Preventar-13 for the prevention of various diseases of infectious etiology in children showed its high efficiency in the prevention of pneumonia. The coefficient of preventive effectiveness of vaccination in most municipalities of the region on the indicator of the incidence of pneumonia has reached 75-100%. It was found that there is a direct dependence of the degree of effectiveness of vaccination against pneumococcal infection on the age of children: the older the child is, the higher the efficiency becomes (r=0.94), at t =6.71, p≤0.05.

One of the main indicators of the effectiveness of prevention programs is the incidence of vaccinated population in pre- and postvaccination periods. The results of the analysis of vaccine used in the Amur region show that the number of pneumonia diseases registered in the observed population decreased by 2.3 times in the postvaccination period (p=0.034).

**Figure 6:**
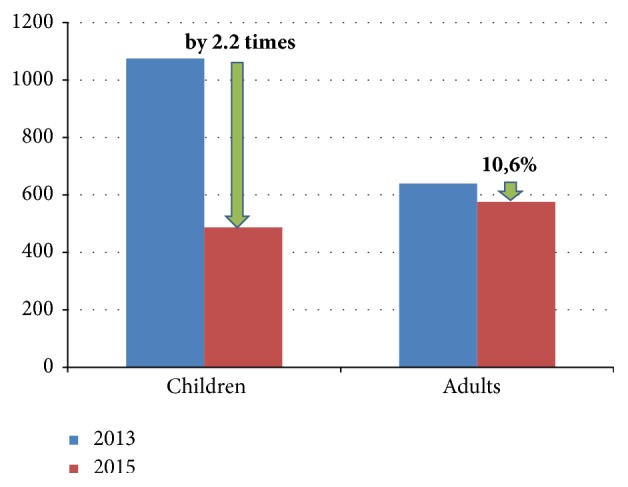
Comparative analysis of dynamics of morbidity of children's and adult populations of the Amur Region (data of the epidemiological monitoring, per 100 000 population).

Analysis of the results of epidemiological monitoring of the incidence of pneumonia in the population of the Amur region shows (Figure 6) that among the child population the rate of decline in morbidity for the period 2013-2015 is significantly higher (2.2 times) than among the adult population (10.6%), p=0.043. This fact demonstrates the effectiveness of using pneumococcal vaccine in preventing pneumococcal infections: vaccine prophylaxis of pneumococcal infections involved the child population. In the postvaccination period, the incidence of pneumonia in children is significantly lower in almost all recorded periods of the year. At the same time, for the first time the incidence of pneumonia in children in the region in 2015 was lower than the incidence of adult population.

Over the past 10 years, the coverage of the population with influenza vaccination has increased 2.2 times (in 2015, 39.7%; in 2005, 17.5%). At the same time, the incidence of ARVI and influenza in the region over the past 6 years decreased by 29.2% (from 22216.9 per 100 000 population in 2009 to 15737.7 in 2015); r=-1.0, p≤0.05.

Analysis of the results of the implementation of preventive programs shows that in general the level of cumulative morbidity of children with ARVI and pneumonia in the postvaccination period (2015) decreased compared to the prevaccination period (2013) by 2.5 times (from 3725.8‰ to 1471.4‰). If in the administrative territories of the region included in the program of vaccine prevention of pneumococcal infections, the incidence of pneumonia decreased among the total population by 25.6%, then in the territories not included in it, this happened only by 0.29% (p≤0.05). Consequently, the use of pneumococcal vaccine had a significant impact on the effectiveness of prevention programmes. During the period of implementation of preventive programs, the incidence of pneumonia in the Amur region decreased by 23.1%, and mortality from these causes by 39.4% (p≤0.05). By reducing the incidence of 1505 cases for the period 2013-2015, an economic damage in the amount of 139.5 million roubles was prevented.

## 4. Conclusions

Currently on the territory of the Far-Eastern region of Russia, the experience of how to organize and provide the complex of antiepidemic and preventive measures aimed at reducing the level of incidence of acute respiratory infections and pneumonia in the conditions of influence of extreme climatic factors is accumulated. Emergency vaccination against pneumococcal infection in the Khabarovsk Krai of Russia led to a decrease in the frequency of nasopharyngeal carriage in vaccinated children of pneumococcus 19F, 4, 6A/BC/D, and 14 serovariants by 1.5-2 times and complete elimination of pneumococcus from the nasopharynx in 32.1% of cases (p<0.05).

The implementation of regional preventive programs in the field of respiratory health care using vaccines against pneumococcal infection has a high level of medical and socioeconomic efficiency. This allows us to recommend its use as the most effective method of prevention of respiratory viral and pneumococcal infections in the complex of antiepidemic and preventive measures among different age groups.

## Data Availability

No data were used to support this study.
